# Patients with metastatic renal cell carcinoma who benefit from axitinib dose titration: analysis from a randomised, double-blind phase II study

**DOI:** 10.1186/s12885-018-5224-6

**Published:** 2019-01-07

**Authors:** Yoshihiko Tomita, Hirotsugu Uemura, Mototsugu Oya, Nobuo Shinohara, Tomonori Habuchi, Yosuke Fujii, Yoichi Kamei, Yoshiko Umeyama, Angel H. Bair, Brian I. Rini

**Affiliations:** 1 Department of Urology, Department of Molecular Oncology, Niigata University Graduate School of Medical and Dental Sciences, Asahimachi 1-757, Niigata, 951-8510 Japan; 2 Department of Urology, Kindai University Faculty of Medicine, Osaka, Japan; 3 Department of Urology, Keio University School of Medicine, Tokyo, Japan; 4 Department of Urology, Hokkaido University Graduate School of Medicine, Sapporo, Hokkaido Japan; 5 Department of Urology, Akita University School of Medicine, Akita, Japan; 6 Pfizer Japan Inc, Tokyo, Japan; 7 Pfizer Oncology, San Diego, CA USA; 8 Department of Solid Tumour Oncology, Cleveland Clinic Taussig Cancer Institute, Cleveland, OH USA

**Keywords:** Axitinib, Benefit with dose titration, First-line, Metastatic renal cell carcinoma, Predictive factors, Survival benefit

## Abstract

**Background:**

A prospective, randomised phase II study demonstrated clinical benefit of axitinib dose titration in a subset of treatment-naïve patients treated with axitinib for metastatic renal cell carcinoma. This analysis evaluated patient baseline characteristics that may impact overall survival (OS) with axitinib dose titration.

**Methods:**

Following a 4-week lead-in period during which all patients received axitinib 5 mg twice-daily (bid); patients meeting the predefined randomisation criteria were randomly assigned to receive axitinib 5 mg bid plus either axitinib or placebo titration. In exploratory analyses, patients were grouped into those who achieved OS ≥24 versus < 24 months, and compared their baseline characteristics with Fisher’s exact test or Cochran-Armitage trend exact test, with a 5% significance level. Potential predictive baseline characteristics associated with effect of axitinib dose titration on OS were investigated using a Cox proportional hazard model.

**Results:**

Overall, 112 patients were randomised. Three of 56 patients receiving axitinib titration were censored; of the remaining 53, 33 (62%) achieved OS ≥24 months versus 20 (38%) with OS < 24 months. Patients with OS ≥24 vs. < 24 months, respectively, had significantly fewer metastatic sites (≤2 metastases: 52% vs. 10%; ≥3 metastases: 48% vs. 90%), fewer lymph node (45% vs. 75%) or liver (15% vs. 45%) metastases, higher haemoglobin level (i.e., ≥ lower limit of normal: 67% vs. 25%) at baseline, lower neutrophil (≤ upper limit of normal, 97% vs. 75%) and platelet (≤ upper limit of normal, 82% vs. 50%) levels at baseline and ≥ 1 year between histopathological diagnosis and treatment (64% vs. 15%). The primary reason for treatment discontinuation in both OS groups was disease progression. The frequency of toxicity-related discontinuation was comparable between the 2 groups, indicating that it was not a factor for a shorter OS. The multivariate analysis showed that ≥1 year from histopathological diagnosis to treatment and baseline haemoglobin level equal or greater than lower limit of normal were significant covariates associated with favourable OS in patients receiving axitinib titration.

**Conclusions:**

The current analyses identified potentially predictive factors that could help selecting patients who may benefit from axitinib dose titration.

**Trial registration:**

ClinicalTrials.gov identifier, NCT00835978. Registered prospectively, February 4, 2009.

**Electronic supplementary material:**

The online version of this article (10.1186/s12885-018-5224-6) contains supplementary material, which is available to authorized users.

## Background

Axitinib is a potent and selective inhibitor of vascular endothelial growth factor (VEGF) receptors 1, 2, and 3 approved for second-line treatment in patients with metastatic renal cell carcinoma (mRCC) [1]. Unlike the majority of other targeted agents for cancer treatment, in which dose increase is often not permitted, axitinib starting dose of 5 mg twice daily (bid) can be increased stepwise to 7 mg bid, and to a maximum of 10 mg bid, to improve efficacy outcomes, if patients tolerate the drug. Conversely, the starting dose can be decreased to manage drug-related toxicity.

**Fig. 1:**
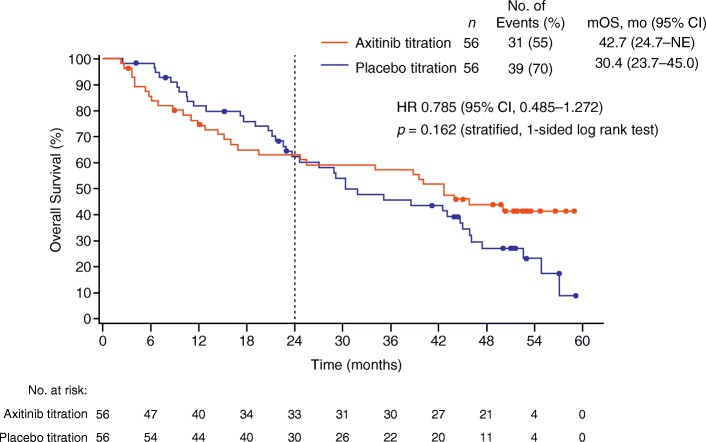
Kaplan-Meier estimates for OS in the axitinib- and placebo-titration arms. Modified from Rini et al. [3], Overall survival analysis from a randomized phase II study of axitinib with or without dose titration in first-line metastatic renal cell carcinoma, p 500, copyright 2016, with permission from Elsevier. *Abbreviations*: *CI* confidence interval, *LLN* lower limit of normal, *mOS* median overall survival, *NE* not estimable, *OS* overall survival

The effect of axitinib dose titration was prospectively evaluated in a global, randomised phase II study in patients with mRCC who had not received any prior systemic therapy for the disease [2]. The study results showed a higher proportion of patients who received axitinib dose titration in addition to the starting 5 mg bid dose achieved objective response compared with those who received placebo titration plus the starting dose (5 mg bid) of axitinib (54% vs. 34%, respectively; 1-sided *p* = 0.019), thus providing evidence for clinical benefit of individualised axitinib dose titration in some patients. A follow-up analysis indicated a numerically longer overall survival (OS) in the axitinib dose titration arm compared with placebo titration arm (median OS, 42.7 vs. 30.4 months; 1-sided *p* = 0.162), without any new safety concerns [3]. It was noted in the study that the Kaplan–Meier curves for OS estimates for the axitinib dose titration and placebo titration arms appeared to cross over at approximately 24 months following treatment initiation (Fig. 1) [3]. This phenomenon might have been attributed to a random occurrence, but it could also have been the summation of various clinical responses following axitinib dose titration. For instance, some patients in the axitinib titration arm might have discontinued treatment early due to drug-related toxicities as a result of aggressive dose increase. It might also be plausible that in patients with poor baseline prognosis, tumours could have rapidly progressed before achieving the therapeutic level of axitinib with dose titration. Whatever the reason(s) may be for the crossover, it is likely that patients who had OS ≥24 months had attained clinical benefits with axitinib dose titration.

The current analysis was conducted to evaluate patient baseline characteristics that may be associated with longer OS in patients treated with axitinib dose titration, which would help identify patients who potentially benefit from axitinib dose titration.

## Methods

### Study design

**Fig. 2:**
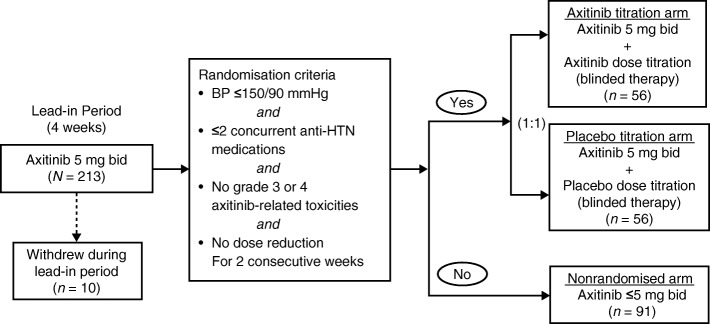
Study design [8]. *Abbreviations*: *bid* twice daily; *BP* blood pressure; *HTN* hypertension

The data used in the current analysis were from the randomised, double-blind phase II study of axitinib dose titration conducted in 6 countries (Czech Republic, Germany, Japan, Russia, Spain and the United States). Details of the study design (Fig. 2) and the results of the primary endpoint, which was comparison of the percentage of patients who achieved an objective response between randomised arms, have been previously published [2].

The study was conducted in accordance with the Declaration of Helsinki, the International Conference on Harmonisation guidelines on Good Clinical Practice, and all applicable local regulations and laws. The study protocol and patient informed consent form were approved by the institutional review board or independent ethics committee at each centre, and each patient provided written informed consent prior to study entry. This trial is registered at ClinicalTrials.gov (identifier NCT00835978).

### Patients and treatment

Inclusion and exclusion criteria have been described in detail elsewhere [2]. In brief, key eligibility included histologically confirmed mRCC with a clear cell component based on laboratory assessments conducted at each site; ≥1 measurable disease by Response Evaluation Criteria in Solid Tumours (RECIST) v1.0; no prior systemic therapy for mRCC; Eastern Cooperative Oncology Group performance status (ECOG PS) 0 or 1; and no uncontrolled hypertension (blood pressure [BP] ≤140/90 mmHg).

All eligible patients received the axitinib 5 mg bid starting dose during a 4-week lead-in period, after which patients were randomly assigned to axitinib or placebo titration if they had met the randomisation criteria for 2 consecutive weeks: BP ≤150/90 mmHg; no grade 3 or 4 drug-related adverse events (AEs), according to the Common Terminology Criteria for Adverse Events v3.0; no axitinib dose reduction; and use of ≤2 concurrent antihypertensive medications. Patients in both titration arms had their daily dose of axitinib or placebo increased by 2 mg bid in a blinded fashion, to a total of 7 mg bid. If patients tolerated the drug at this dose by meeting the randomisation criteria, the dose could be further increased to the maximum total dose of 10 mg bid (including the axitinib 5-mg starting dose). Patients who did not meet the randomisation criteria continued treatment in a nonrandomised arm.

### Assessments and statistical analyses

Radiologic tumour assessments were performed by investigators at baseline, weeks 8, 16, and 24, and every 12 weeks thereafter, according to RECIST. Survival data were collected every 3 months after the last follow-up visit, which was 28 days after the last dose.

In exploratory analyses, patients in the axitinib or placebo titration arm were grouped into those with a longer OS (i.e., OS ≥24 months) versus shorter OS (i.e., OS < 24 months). A cutoff of 24 months was chosen because it was the time period at which there was a crossover of the OS curves between the axitinib and placebo titration arms. Demographics and baseline characteristics of patients who with OS ≥24 months versus < 24 months in the axitinib or placebo titration arm were compared with Fisher’s exact test for binary categorical variables or Cochran-Armitage trend exact test for ordinal categorical variables, with a 5% significance level.

For selected baseline characteristics, a multivariate analysis using a Cox proportional hazard model was subsequently conducted to investigate the potential predictive baseline characteristics associated with the effect of axitinib dose titration on OS. This analysis was performed for all randomised patients in both the axitinib and placebo titration arms to evaluate the interaction effects between axitinib/placebo titration and baseline characteristics on OS. Median OS and 95% confidence intervals (CIs) for potential predictive baseline characteristics were estimated using the Kaplan–Meier method.

## Results

### Patients

Of 112 patients who met the randomisation criteria, 56 each were randomly assigned to the axitinib and placebo titration arms. Patient disposition in the randomised arms is summarised in Table 1.

**Table 1:** Patient disposition in the axitinib and placebo titration arms

Disposition, *n* (%)	Axitinib Titration *n* = 56	Placebo Titration *n* = 56
Discontinued treatment	47 (84)	55 (98)
Disease progression/relapse	35 (63)	40 (71)
Adverse event	8 (14)	5 (9)
Death	2 (4)	1 (2)
Refusal of treatment for reason other than adverse event	1 (2)	4 (7)
Global deterioration in health status	1 (2)	1 (2)
Protocol violation	0	1 (2)
Other	0	3 (5)
Remaining on treatment	9 (16)	1 (2)

In the axitinib titration arm, 3 patients who were censored < 24 months in OS, were excluded from the dataset; the remaining 53 patients were included in the analysis. Of these, 33 (62%) patients achieved OS ≥24 months, whereas 20 (38%) had OS < 24 months. Comparison of demographics and baseline characteristics between axitinib-titrated patients who achieved OS ≥24 versus < 24 months, respectively, showed that patients with a longer OS had significantly fewer metastatic sites (≤2 metastases: 52% vs. 10%; ≥3 metastases: 48% vs. 90%), fewer lymph node (45% vs. 75%) or liver (15% vs. 45%) metastases at baseline, longer time between histopathological diagnosis and treatment (≥1 year: 64% vs. 15%), higher than lower limit of normal (LLN) baseline haemoglobin levels (67% vs. 25%), lower than upper limit of normal (ULN) baseline neutrophil levels (97% vs. 75%), and lower than ULN baseline platelet levels (82% vs. 50%) (Table 2). Although a higher percentage of patients with OS ≥24 months relative to those with OS < 24 months received prior nephrectomy (97% vs. 80%) or had fewer bone metastases (6% vs. 25%) at baseline, the difference did not reach a statistical significance. There was no significant difference in baseline ECOG PS, histological classification, or tumour size between the 2 groups (Table 2).

**Table 2:** Baseline characteristics of patients with OS ≥24 versus < 24 months in the axitinib titration arm

Baseline Characteristics, *n* (%)		OS ≥24 months *n* = 33	OS < 24 months *n* = 20^a^	*p*-value^b^
Age, years	< 65	19 (58)	17 (85)	0.0670
	≥65	14 (42)	3 (15)	
Sex	Male	21 (64)	15 (75)	0.5457
	Female	12 (36)	5 (25)	
Race	White	28 (85)	18 (90)	0.2672
	Asian	5 (15)	1 (5)	
	Other	0	1 (5)	
Weight, kg	≤65	7 (21)	5 (25)	1.0000
	> 65, ≤76	12 (36)	7 (35)	
	> 76, ≤89	8 (24)	3 (15)	
	> 89	6 (18)	5 (25)	
ECOG PS	0	23 (70)	11 (55)	0.3775
	≥1	10 (30)	9 (45)	
Histological classification	Clear cell	33 (100)	18 (90)	0.1379
	Other^c^	0	2 (10)	
Prior nephrectomy	Yes	32 (97)	16 (80)	0.0611
	No	1 (3)	4 (20)	
No. of metastatic sites	≤2	17 (52)	2 (10)	0.0028
	≥3	16 (48)	18 (90)	
Site of metastasis	Lung only	4 (12)	0	0.2848
	Lung + others	29 (88)	20 (100)	
Site of metastasis, individual				
	Lung	19 (58)	16 (80)	0.1368
	Lymph node	15 (45)	15 (75)	0.0476
	Kidney	7 (21)	4 (20)	1.0000
	Liver	5 (15)	9 (45)	0.0252
	Adrenal	10 (30)	4 (20)	0.5274
	Bone	2 (6)	5 (25)	0.0896
	Pancreas	2 (6)	0	0.5210
Time from histopathological diagnosis to treatment				
	≥1 year	21 (64)	3 (15)	0.0007
	< 1 year	12 (36)	17 (85)	
Time from metastatic diagnosis to treatment				
	≥1 year	4 (12)	1 (5)	0.6388
	< 1 year	29 (88)	19 (95)	
Sum of longest diameter for target lesion				
	≤Median	17 (52)	7 (35)	0.2702
	>Median	16 (48)	13 (65)	
Presence of metastases (de novo) at initial diagnosis				
	No	22 (67)	12 (60)	0.7689
	Yes	11 (33)	8 (40)	
Baseline LDH	≤1.5 × ULN	33 (100)	18 (90)	0.1379
	> 1.5 × ULN	0	2 (10)	
Baseline Hb	≥LLN	22 (67)	5 (25)	0.0047
	<LLN	11 (33)	15 (75)	
Baseline Neu	≤ULN	32 (97)	15 (75)	0.0240
	>ULN	1 (3)	5 (25)	
Baseline Plt	≤ULN	27 (82)	10 (50)	0.0288
	>ULN	6 (18)	10 (50)	

*Abbreviations*: *ECOG PS* Eastern Cooperative Oncology Group performance status, *Hb* haemoglobin, *LDH* lactate dehydrogenase, *LLN* lower limit of normal, *Neu* neutrophil, *OS* overall survival, *Plt* platelet, *ULN* upper limit of normal

^a^Excluded 3 patients who were censored <24 months in OS

^b^Fisher’s exact test was used for all, except weight, for which Cochran-Armitage trend test was used

^c^Other includes sarcomatoid and clear cell with sarcomatoid differentiation in 1 patient each

In the placebo titration arm, 6 patients were censored < 24 months in OS and excluded from the analysis; of the remaining 50 patients, 30 (60%) achieved OS ≥24 months, and 20 (40%) had OS < 24 months. No significant difference was observed in demographics or baseline characteristics of placebo-titrated patients with OS ≥24 versus < 24 months in the placebo titration arm (Additional file 1).

### Treatment

Patients in the axitinib titration arm with OS ≥24 versus < 24 months, respectively, received comparable average daily doses of axitinib (median: 13.9 vs. 13.0 mg) and median relative dose intensity was similar (135% vs. 125%) (Table 3). As expected, patients who achieved OS ≥24 months received axitinib treatment for a significantly longer period than those with OS < 24 months. Median number of days from date of the first dose to date of the last dose or data cutoff was 925 days in patients with OS ≥24 months versus 85 days in patients with OS < 24 months; median number of days in which axitinib was administered was 925 and 84 days, respectively. In the placebo titration arm, patients with OS ≥24 months also received treatment for a longer period than those with OS < 24 months (Additional file 2).

**Table 3:** Drug exposure in patients with OS ≥24 versus < 24 months in the axitinib titration arm

Parameter	OS ≥24 months *n* = 33	OS < 24 months *n* = 20^a^
Days on treatment,^b^ median (range)	925 (106–1795)	85 (42–456)
Days on drug,^c^ median (range)	925 (91–1688)	84 (38–456)
Average daily dose, median (range), mg	13.9 (4.9–23.4)	13.0 (9.9–19.2)
Relative dose intensity, median (range), %	135 (46–224)	125 (83–192)
Patients with dose decrease < 5 mg bid, *n* (%)	10 (30)	1 (5)

*Abbreviations*: *bid* twice daily, *OS* overall survival

^a^Excluded 3 patients who were censored < 24 months in OS

^b^Time period starting from date of the first dose to date of the last dose or data cutoff

^c^Total number of days in which axitinib was actually administered

At the data cutoff date, 24 (73%) and 20 (100%) axitinib-titrated patients who achieved OS ≥24 versus < 24 months, respectively, had discontinued treatment. Although the primary reason for treatment discontinuation was disease progression, it is notable that the percentage of patients who progressed was lower in the OS ≥24 versus < 24 months groups (52% vs. 80%, respectively; Table 4). The frequency of discontinuation due to AEs was generally comparable between OS ≥24 and < 24 months (15% vs. 10%). Reasons for treatment discontinuation in the placebo titration arm were similar to those in the axitinib titration arm, with disease progression being the primary reason (Additional file 3).

**Table 4:** Reasons for treatment discontinuation of patients with OS ≥24 versus < 24 months in the axitinib titration arm

Reason for treatment discontinuation, *n* (%)	OS ≥24 months *n* = 33^a^	OS < 24 months *n* = 20^b^
Objective progression or relapse	17 (52)	16 (80)
Adverse event	5 (15)	2 (10)
Death	1 (3)	1 (5)
Global deterioration of health status	0	1 (5)
Patient refusal to continue treatment for reason other than adverse event	1 (3)	0
Total	24 (73)	20 (100)

*Abbreviation*: *OS* overall survival

^a^Including 9 patients still on treatment

^b^Excluded 3 patients who were censored < 24 months in OS

### Safety

In the axitinib titration arm, the common treatment-emergent, all-causality, all-grade AEs reported by > 50% of patients included diarrhoea, hypertension, and fatigue in the OS ≥24-months group, and hypertension and nausea in the OS < 24-months group (Table 5). The nature of AEs reported by patients with OS ≥24 months was similar to those with OS < 24 months, but incidence rates of diarrhoea, hypertension, hypothyroidism, hand–foot syndrome, and stomatitis were substantially (> 20%) higher in patients with OS ≥24 months than OS < 24 months. Hypertension was the frequently reported grade 3 or 4 AE in either group. The nature and severity of AEs in patients with OS ≥24 and OS < 24 months in the placebo titration arm were generally similar to those in the corresponding OS groups in the axitinib titration arm (Additional file 4).

**Table 5:** Common, all-causality adverse events reported in patients with OS ≥24 versus < 24 months in the axitinib titration arm

Adverse event,^a^ *n* (%)	OS ≥24 months *n* = 33		OS < 24 months *n* = 20^b^	
	All Grades	Grade ≥ 3	All Grades	Grade ≥ 3
Any	33 (100)	24 (73)	19 (95)	11 (55)
Diarrhoea	27 (82)	7 (21)	7 (35)	0
Hypertension	24 (73)	7 (21)	10 (50)	3 (15)
Fatigue	18 (55)	1 (3)	9 (45)	2 (10)
Nausea	14 (42)	1 (3)	10 (50)	2 (10)
Hand–foot syndrome	14 (42)	1 (3)	4 (20)	1 (5)
Hypothyroidism	14 (42)	0	4 (20)	0
Decreased appetite	12 (36)	2 (6)	8 (40)	2 (10)
Weight decrease	12 (36)	6 (18)	4 (20)	0
Vomiting	11 (33)	3 (9)	8 (40)	1 (5)
Dysphonia	11 (33)	0	7 (35)	1 (5)
Arthralgia	10 (30)	0	3 (15)	1 (5)
Stomatitis	10 (30)	1 (3)	0	0
Mucosal inflammation	9 (27)	1 (3)	3 (15)	1 (5)
Proteinuria	9 (27)	2 (6)	3 (15)	0

*Abbreviation*: *OS* overall survival

^a^Reported by ≥25% in either group

^b^Excluded 3 patients who were censored < 24 months in OS

### Predictive factors for survival benefit with axitinib dose titration

In the multivariate analysis conducted using the data from all 112 randomised patients, time from histopathological diagnosis to treatment ≥1 year and baseline haemoglobin level ≥ LLN were found to be independent and significant (*p* < 0.1 for interaction) predictive covariates associated with favourable OS in the axitinib titration arm, whereas a trend for association was observed with the number of metastatic sites (Table 6). Baseline neutrophil and platelet levels were excluded from the multivariate analysis due to the limited number of patients with >ULN. The presence of ≤2 metastatic sites was associated with longer OS with axitinib titration and placebo titration (hazard ratio [HR] 2.191 and 4.438; *p* = 0.0179), but the interaction was not significant (*p* = 0.2321, Additional file 5). There was no difference for OS between < 1 versus ≥1 year from histopathological diagnosis to treatment with placebo titration (HR 1.437; *p* = 0.3218), whereas there was significant interaction with axitinib titration (HR 3.569; *p* = 0.0900 for interaction). Similarly, baseline haemoglobin level ≥ LLN was associated with longer OS in patients receiving axitinib titration (HR 3.378; *p* = 0.0126 for interaction), but not in patients in the placebo titration arm (HR 0.940; *p* = 0.8543) (Additional file 5).

**Table 6:** Multivariate analysis of patient baseline characteristics for the effect of interaction with axitinib titration on OS

Parameter	HR for Covariate	
	Placebo Titration	Axitinib Titration
Metastatic site (≥3 vs. ≤2)	2.191	4.438
Time from histopathological diagnosis to treatment (< 1 vs. ≥1 year)	1.437	3.569
Baseline Hb < LLN (yes vs. no)	0.940	3.378

*Abbreviations*: *CI* confidence interval, *Hb* haemoglobin, *HR* hazard ratio, *LLN* lower limit of normal

**Fig. 3:**
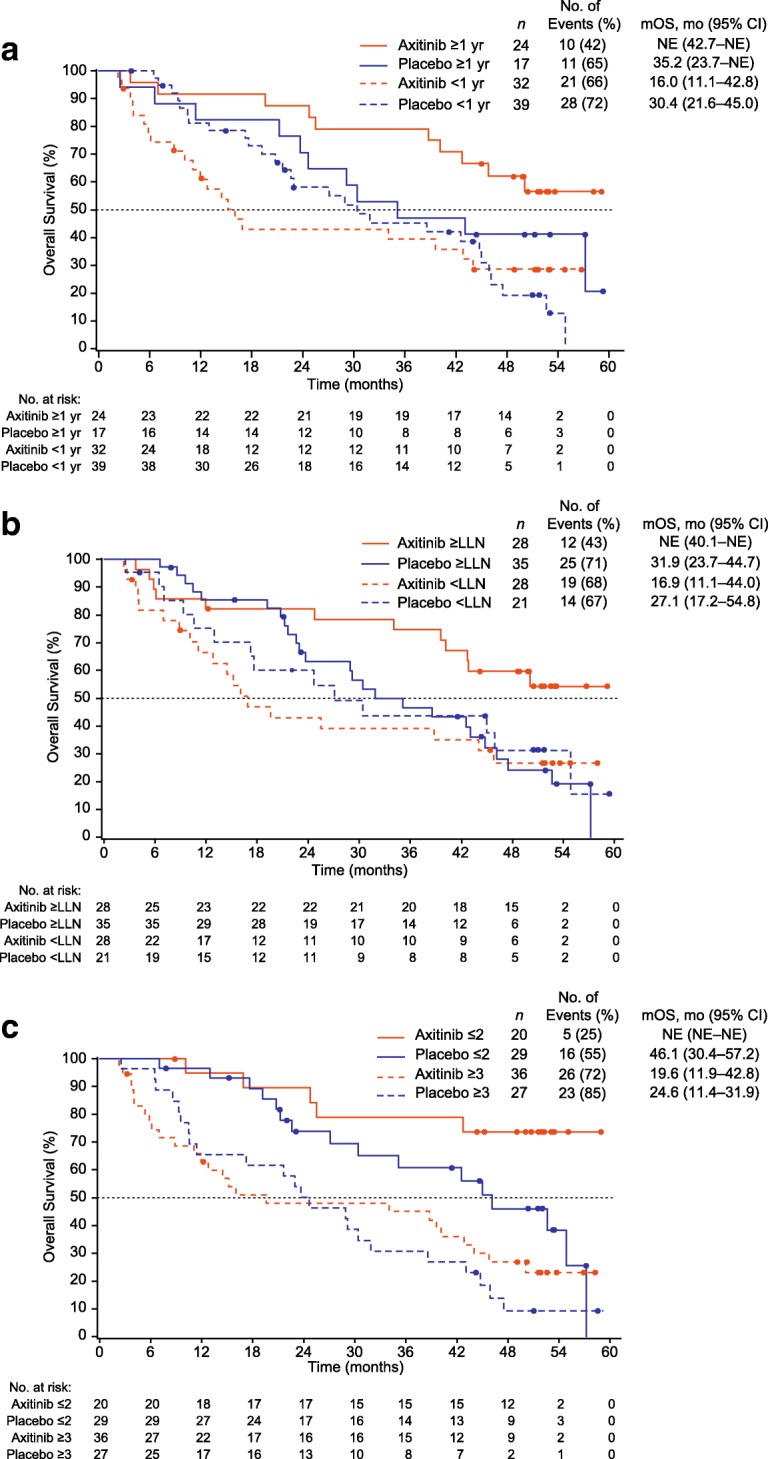
Kaplan–Meier estimates for OS in the axitinib- and placebo-titration arms. (**a**) Stratified by time from histopathological diagnosis to treatment, (**b**) stratified by baseline haemoglobin level, and (**c**) stratified by number of metastatic sites. *Abbreviations*: *CI* confidence interval, *LLN* lower limit of normal, *mOS* median overall survival, *NE* not estimable, *OS* overall survival

To visually evaluate the potential effect of axitinib dose titration on each covariate for OS (i.e., predictive value of each covariate for OS), median OS was estimated in the axitinib and placebo titration arms following stratification by the individual covariate identified as significant in the multivariate analysis. Median follow-up for OS in all 112 randomised patients was 30.4 months. In the axitinib titration arm, median OS (95% CI) was not estimable (42.7 months–not estimable) in patients with ≥1 year between histopathological diagnosis and treatment compared with 16.0 (11.1–42.8) months in patients treated < 1 year post diagnosis (Fig. 3a), whereas in the placebo titration arm, median OS was 35.2 (23.7–not estimable) versus 30.4 (21.6–45.0) months (≥1 vs. < 1 year, respectively).

Similarly, in patients with ≥LLN baseline haemoglobin level, median OS (95% CI) in the axitinib titration arm was not estimable (40.1 months–not estimable) compared with 16.9 (11.1–44.0) months in those with <LLN baseline levels (Fig. 3b), whereas no notable difference was found in patients in the placebo titration arm: 31.9 (23.7–44.7) vs. 27.1 (17.2–54.8) months. When stratified by number of metastatic sites, median OS (95% CI) in patients in the axitinib titration arm who had ≤2 metastatic sites was not estimable (not estimable–not estimable) compared with 19.6 (11.9–42.8) months in patients with ≥3 metastatic sites. In the placebo titration arm, patients with ≤2 metastatic sites also had a longer median OS than those with ≥3 metastatic sites: 46.1 (30.4–57.2) vs. 24.6 (11.4–31.9) months, respectively (Fig. 3c).

**Fig. 4:**
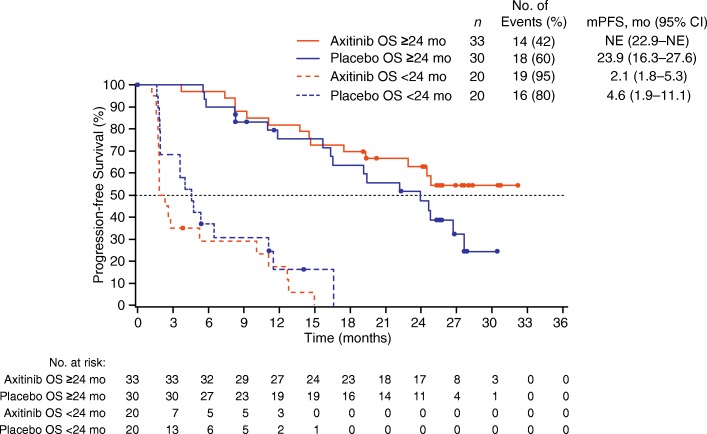
Kaplan–Meier estimates for PFS in the axitinib- and placebo-titration arms stratified by OS. Patients were stratified by OS ≥24 vs. < 24 months. *Abbreviations*: *CI* confidence interval, *mPFS* median progression-free survival, *NE* not estimable, *OS* overall survival

Not surprisingly, in both the axitinib and placebo titration arms, median progression-free survival (PFS) was shorter in patients with OS < 24 months than in patients with OS ≥24 months (Fig. 4).

## Discussion

With several VEGF- and other molecular-targeted agents, including axitinib, which are available as monotherapy and/or in sequential therapy, survival rates for patients with mRCC have drastically increased in the past decade. However, not all patients benefit from these targeted agents and, therefore, identification and validation of biomarkers that may facilitate selection of patients who would achieve clinical benefit from individual targeted agents has become one of the critical challenges in the treatment algorithm for mRCC. Although several studies have reported a number of patient baseline characteristics as potential biomarkers for PFS and/or OS in patients with mRCC treated with targeted therapy [4–7], none to date have been validated for use in a routine clinical setting. This study investigated patient baseline characteristics that may render survival benefit with axitinib dose titration in the first-line setting for patients with mRCC.

The current analyses revealed several interesting findings. Among 56 treatment-naïve patients with mRCC who were randomly assigned to the axitinib titration arm, 33 attained OS ≥24 months and 20 did not. Patients with OS ≥24 months had more favourable baseline characteristics and received a significantly longer duration of treatment than patients with OS < 24 months. The discontinuations due to AEs were not a factor for the shorter OS since the frequency of AE-related discontinuations was comparable between the 2 groups. Although the incidence of diarrhoea, hypertension, hypothyroidism, hand–foot syndrome, and stomatitis were substantially greater in patients with OS ≥24 months than OS < 24 months, this may be a reflection of the longer treatment duration that patients with OS ≥24 months received compared with patients with OS < 24 months. It should be pointed out that in the OS < 24 months group, more than 50% of patients developed disease progression by approximately 3 months, leading to a shorter treatment duration in this group.

In the multivariate analysis, time from histopathological diagnosis to treatment ≥1 year was associated with longer OS with axitinib titration, whereas there was no significant difference between < 1 versus ≥1 year with placebo titration. Similarly, baseline haemoglobin level ≥ LLN was associated with longer OS in patients receiving axitinib titration, but not in patients in the placebo titration arm. These results suggest that time between diagnosis and treatment and baseline haemoglobin level may potentially serve as predictive baseline characteristics for OS with axitinib titration. As for the number of metastatic sites, OS was different between patients with ≥3 versus ≤2 metastatic sites at baseline in the axitinib titration arm, but this trend was also seen in the placebo titration arm, indicating that number of metastatic sites is a prognostic factor for OS, although it potentially has a predictive value for OS with axitinib titration.

Tomita and his colleagues previously reported the results of a multivariate analysis for predictive factors of PFS, which was based on the same dose titration study but used data from all 213 patients enrolled in the study [8]. That analysis identified time from diagnosis to treatment and baseline tumour size as independent predictive factors for PFS. In another multivariate analysis, Oya et al. identified longer time from histopathological diagnosis to treatment, lower baseline tumour burden, and lower baseline ECOG PS as positive predictors of OS [9]. Time from diagnosis to treatment was the common predictive factor for OS found by both Oya et al. and the current analysis, whereas baseline tumour burden and ECOG PS were unique to the analysis by Oya et al. and baseline haemoglobin level was unique to the current analysis. A possible reason for differences in factors predictive of better OS or PFS among the 3 analyses may be attributable to the difference in the datasets used, i.e., 213 patients, including 10 patients who discontinued prior to randomisation as well as 91 patients who were not randomised, in the previous 2 analyses compared with 112 randomised patients in the current analysis. It may also be explained, at least in part, by the methodological differences: the current analysis evaluated the effect of axitinib versus placebo titration on OS and, additionally, investigated the effect of interaction between axitinib dose titration and the covariate effect on OS, which was not conducted previously. The predictive factors identified for clinical benefit of axitinib in these 3 analyses are not completely the same, but all are related to better prognosis for patients at baseline. Therefore, it has been postulated that patients with more favourable baseline characteristics would preferentially receive PFS and OS benefit from axitinib treatment and OS benefit from axitinib dose titration.

The Memorial Sloan Kettering Cancer Center (MSKCC) and the International Metastatic Renal-Cell Carcinoma Database Consortium (IMDC) risk factors have been established and validated for classification of baseline prognosis in patients with mRCC treated with cytokines or VEGF-targeted agents [4–7]. Many of these MSKCC and IMDC risk factors have been confirmed in the multivariate analysis for baseline prognostic factors for OS in the phase III trial of axitinib in patients previously treated with systemic therapy for mRCC [10]. In that analysis, time from diagnosis to treatment and baseline haemoglobin level, which were identified in our analysis, were found among the prognostic factors. With regard to haemoglobin, it is worth mentioning that in a retrospective multivariate analysis conducted in 1463 Japanese patients with mRCC, a low baseline level of haemoglobin was correlated with a high baseline level of C-reactive protein, and the high level of C-reactive protein at the metastatic diagnosis was significantly associated with poor prognosis [11]. Although that analysis was performed using data from the cytokine era, similar observations regarding the potential prognostic value of C-reactive protein have been reported in patients with mRCC treated with targeted therapies [12, 13].

It is interesting that in the validation study for the MSKCC risk criteria, Motzer and his colleagues also analysed baseline characteristics predictive of long-term OS, defined as OS ≥30 months, in sunitinib-treated patients with mRCC [6]. A number of baseline characteristics, including ethnic origin, MSKCC risk factors, ECOG PS, and prior nephrectomy, differed significantly between patients with long-term OS (*n* = 215) and non–long-term OS (*n* = 844). The multivariate analysis found ethnic origin, baseline bone metastases, and corrected calcium levels to be independent prognostic factors in patients with long-term OS. Possible reasons for the differences between the results by Motzer et al. and the current analysis may be the difference in sample size and/or time used as a cutoff, but it may also be the difference in the methods utilised for the statistical analysis: in the current analysis, the effect of interaction with axitinib dose titration versus placebo titration was investigated.

It had been speculated that aggressive axitinib dose increases might result in increased drug-related toxicities and, consequently, early treatment discontinuation. However, our analyses uncovered that the shorter OS was not due to toxicity-induced early treatment discontinuation, but rather likely to patient baseline characteristics. Up until now it has been unclear whether there are any baseline patient characteristics other than the dose titration criteria that were applied to axitinib clinical trials that should be considered when selecting patients for axitinib treatment with dose titration. The results of the current analyses may be helpful for physicians to potentially determine prospective patients who may receive clinical benefit from axitinib dose titration.

The current study had some limitations. The first limitation was the retrospective exploratory nature of the current analysis. In addition, the baseline patient characteristics which were not stratification factors in the original study were evaluated. Another is that in the current study, calcium concentration, which is one of the MSKCC risk factors in mRCC, was not collected and, hence, its impact on OS in patients with titrated axitinib doses could not be evaluated. Also, due to the relatively small sample size, the current analyses lacked the power to draw definitive conclusions. The current analysis included the evaluation of the effect of axitinib versus placebo titration on OS and thus, factors identified here are potentially predictive of survival with axitinib dose titration.

## Conclusions

The exploratory analysis of the data from the randomised phase II study of axitinib titration in treatment-naïve patients with mRCC found that patients with ≥1 year from histopathological diagnosis to treatment and ≥ LLN baseline haemoglobin level may potentially achieve survival benefit from the axitinib dose titration. These proposed baseline characteristics predictive of OS may help identify patients with mRCC who would likely benefit from axitinib dose titration.

## Additional files

Additional file 1: Table presenting “Baseline characteristics of patients with OS ≥24 versus <24 months in the placebo titration arm.” (PDF 116 kb)

Additional file 2: Table presenting “Drug exposure in patients with OS ≥24 versus <24 months in the placebo titration arm.” (PDF 65 kb)

Additional file 3: Table presenting “Reasons for treatment discontinuation of patients with OS ≥24 versus <24 months in the placebo titration arm.” (PDF 65 kb)

Additional file 4: Table presenting “Common, all-causality adverse events reported in patients with OS ≥24 versus <24 months in the placebo titration arm.” (PDF 73 kb)

Additional file 5: Table presenting “Multivariate analysis of patient baseline characteristics for the effect of interaction with axitinib titration on OS.” (PDF 105 kb)

## Abbreviations

AE: Adverse event
bid: Twice daily
BP: Blood pressure
CI: Confidence interval
ECOG PS: Eastern Cooperative Oncology Group performance status
HR: Hazard ratio
LLN: Lower limit of normal
mRCC: Metastatic renal cell carcinoma
OS: Overall survival
PFS: Progression-free survival
RECIST: Response Evaluation Criteria in Solid Tumours
ULN: Upper limit of normal
VEGF: Vascular endothelial growth factor
