# Supplementary material for: Patients with metastatic renal cell carcinoma who benefit from axitinib dose titration: analysis from a randomised, double-blind phase II study
Source: BMC Cancer. 2019 Jan 7;19:17. doi: 10.1186/s12885-018-5224-6 (PMC6322336; doi:10.1186/s12885-018-5224-6)
Supplement: Supplementary file 1 — Table presenting “Baseline characteristics of patients with OS ≥24 versus <24 months in the placebo titration arm.” (PDF 116 kb) [file 12885_2018_5224_MOESM1_ESM.pdf]

Additional file 1. Baseline characteristics of patients with OS ≥24 versus <24 months in the placebo titration arm

Baseline Characteristics, *n* (%)		OS ≥24 months<br>*n* = 30	OS <24 months<br>*n* = 20[a]	*p*-value[b]
Age, years	<65	19 (63)	15 (75)	0.5382
	≥65	11 (37)	5 (25)	
Sex	Male	25 (83)	14 (70)	0.3109
	Female	5 (17)	6 (30)	
Race	White	26 (87)	18 (90)	1.0000
	Asian	3 (10)	2 (10)	
	Other	1 (3)	0	
Weight, kg	≤65	4 (13)	6 (30)	0.2321
	>65, ≤76	6 (20)	5 (25)	
	>76, ≤89	12 (40)	4 (20)	
	>89	8 (27)	5 (25)	
ECOG PS	0	19 (63)	10 (50)	0.3927
	≥1	11 (37)	10 (50)	
Histological classification	Clear cell	30 (100)	20 (100)	NA
	Other	0	0	
Prior nephrectomy	Yes	22 (73)	17 (85)	0.4895
	No	8 (27)	3 (15)	
No. of metastatic sites	≤2	17 (57)	7 (35)	0.1588
	≥3	13 (43)	13 (65)	
Site of metastasis	Lung only	4 (13)	0	0.1400
	Lung + others	26 (87)	20 (100)	
Site of metastasis, individual				
	Lung	20 (67)	14 (70)	1.0000
	Lymph node	14 (47)	12 (60)	0.3991
	Kidney	10 (33)	5 (25)	0.7536
	Liver	5 (17)	8 (40)	0.1004
	Adrenal	7 (23)	7 (35)	0.5216
	Bone	4 (13)	7 (35)	0.0902

	Pancreas	0	0	NA
Time from histopathological diagnosis to treatment				
	≥1 year<br><1 year	12 (40)<br>18 (60)	5 (25)<br>15 (75)	0.3654
Time from metastatic diagnosis to treatment				
	≥1 year<br><1 year	2 (7)<br>28 (93)	2 (10)<br>18 (90)	1.0000
Sum of longest diameter for target lesion[c]				
	≤Median<br>>Median	14 (48)<br>15 (52)	7 (35)<br>13 (65)	0.3942
Presence of metastases (de novo) at initial diagnosis				
	No<br>Yes	16 (53)<br>14 (47)	6 (30)<br>14 (70)	0.1480
Baseline LDH	≤1.5 × ULN<br>>1.5 × ULN	28 (93)<br>2 (7)	19 (95)<br>1 (5)	1.0000
Baseline Hb	≥LLN<br><LLN	19 (63)<br>11 (37)	12 (60)<br>8 (40)	1.0000
Baseline Neu	≤ULN<br>>ULN	28 (93)<br>2 (7)	15 (75)<br>5 (25)	0.1003
Baseline Plt	≤ULN<br>>ULN	29 (97)<br>1 (3)	17 (85)<br>3 (15)	0.2885

Abbreviations: *ECOG PS* Eastern Cooperative Oncology Group performance status, *Hb* haemoglobin, *LDH* lactate dehydrogenase, *LLN* lower limit of normal, *NA* not applicable *Neu* neutrophil, *OS* overall survival, *Plt* platelet, *ULN* upper limit of normal

[a] Excluded 6 patients who were censored <24 months in OS.

[b] Fisher's exact test was used for all, except weight, for which Cochran-Armitage trend test was used.

[c] 1 patient in the OS≥24 months group did not have any measurable target lesion; hence, was not included in the analysis.
