# Supplementary material for: Patients with metastatic renal cell carcinoma who benefit from axitinib dose titration: analysis from a randomised, double-blind phase II study
Source: BMC Cancer. 2019 Jan 7;19:17. doi: 10.1186/s12885-018-5224-6 (PMC6322336; doi:10.1186/s12885-018-5224-6)
Supplement: Supplementary file 2 — Table presenting “Drug exposure in patients with OS ≥24 versus <24 months in the placebo titration arm.” (PDF 65 kb) [file 12885_2018_5224_MOESM2_ESM.pdf]

Additional file 2. Drug exposure in patients with OS ≥24 versus <24 months in the placebo titration arm

Parameter	OS ≥24 months<br>*n* = 30	OS <24 months<br>*n* = 20[a]
Days on treatment,[b] median (range)	677 (177–1554)	171 (43–505)
Days on drug,[c] median (range)	664 (167–1554)	165 (43–484)
Average daily dose,[d] median (range), mg	18.3 (7.4–19.8)	13.8 (8.0–18.9)
Relative dose intensity, median (range), %	180 (65–198)	135 (77–189)
Patients with dose decrease <5 mg bid, *n* (%)	3 (10)	2 (10)

Abbreviations: *bid* twice daily, *OS* overall survival

[a] Excluded 6 patients who were censored <24 months in OS.

[b] Time period starting from date of the first dose to date of the last dose or data cutoff.

[c] Total number of days in which axitinib was actually administered.

[d] Included placebo titration.
