# Supplementary material for: Patients with metastatic renal cell carcinoma who benefit from axitinib dose titration: analysis from a randomised, double-blind phase II study
Source: BMC Cancer. 2019 Jan 7;19:17. doi: 10.1186/s12885-018-5224-6 (PMC6322336; doi:10.1186/s12885-018-5224-6)
Supplement: Supplementary file 3 — Table presenting “Reasons for treatment discontinuation of patients with OS ≥24 versus <24 months in the placebo titration arm.” (PDF 65 kb) [file 12885_2018_5224_MOESM3_ESM.pdf]

Additional file 3. Reasons for treatment discontinuation of patients with OS ≥24 versus <24 months in the placebo titration arm

Reason for treatment discontinuation, *n* (%)	OS ≥24 months<br>*n* = 30[a]	OS <24 months<br>*n* = 20[b]
Objective progression or relapse	22 (73)	14 (70)
Adverse event	2 (7)	3 (15)
Death	1 (3)	0
Global deterioration of health status	0	1 (5)
Patient refusal to continue treatment for reason other than adverse event	2 (7)	1 (5)
Others	2 (7)	1 (5)
Total	29 (97)	20 (100)

Abbreviation: *OS* overall survival

[a] Including 1 patients still on treatment.

[b] Excluded 6 patients who were censored <24 months in OS.
