# Supplementary material for: Patients with metastatic renal cell carcinoma who benefit from axitinib dose titration: analysis from a randomised, double-blind phase II study
Source: BMC Cancer. 2019 Jan 7;19:17. doi: 10.1186/s12885-018-5224-6 (PMC6322336; doi:10.1186/s12885-018-5224-6)
Supplement: Supplementary file 4 — Table presenting “Common, all-causality adverse events reported in patients with OS ≥24 versus <24 months in the placebo titration arm.” (PDF 73 kb) [file 12885_2018_5224_MOESM4_ESM.pdf]

Additional file 4. Common, all-causality adverse events reported in patients with OS ≥24 versus <24 months in the placebo titration arm

Adverse event,[a] *n* (%)	OS ≥24 months, *n* = 30		OS <24 months, *n* = 20[b]	
	All Grades	Grade ≥3	All Grades	Grade ≥3
Any	30 (100)	14 (47)	17 (85)	12 (60)
Diarrhoea	24 (80)	3 (10)	8 (40)	0
Hypertension	15 (50)	3 (10)	7 (35)	2 (10)
Fatigue	15 (50)	2 (7)	9 (45)	0
Nausea	14 (47)	0	3 (15)	0
Hand–foot syndrome	8 (27)	1 (3)	2 (10)	0
Hypothyroidism	8 (27)	0	5 (25)	0
Decreased appetite	10 (33)	0	6 (30)	0
Weight decrease	8 (27)	3 (10)	4 (20)	0
Vomiting	8 (27)	0	2 (10)	0
Dysphonia	14 (47)	0	4 (20)	0
Arthralgia	9 (30)	2 (7)	2 (10)	0
Proteinuria	9 (30)	0	2 (10)	0
Headache	11 (37)	1 (3)	1 (5)	0

Abbreviation: *OS* overall survival

[a] Reported by ≥25% in either group.

[b] Excluded 6 patients who were censored <24 months in OS.
