# Supplementary material for: Patients with metastatic renal cell carcinoma who benefit from axitinib dose titration: analysis from a randomised, double-blind phase II study
Source: BMC Cancer. 2019 Jan 7;19:17. doi: 10.1186/s12885-018-5224-6 (PMC6322336; doi:10.1186/s12885-018-5224-6)
Supplement: Supplementary file 5 — Table presenting “Multivariate analysis of patient baseline characteristics for the effect of interaction with axitinib titration on OS.” (PDF 105 kb) [file 12885_2018_5224_MOESM5_ESM.pdf]

Additional file 5. Multivariate analysis of patient baseline characteristics for the effect of interaction with axitinib titration on OS

Parameter	Titration	Covariate	Interaction	HR for Covariate	
	HR (95% CI) *p*-value[a]	HR (95% CI) *p*-value[a]	HR (95% CI) *p*-value[a]	Placebo Titration	Axitinib Titration[b]
Metastatic site (≥3 vs. ≤2)	0.131 (0.034–0.510) *p*=0.0033	2.191 (1.145–4.193) *p*=0.0179	2.026 (0.636–6.449) *p*=0.2321	2.191	4.438
Time from histopathological diagnosis to treatment (<1 vs. ≥1 year)		1.437 (0.701–2.943) *p*=0.3218	2.484 (0.868–7.112) *p*=0.0900	1.437	3.569
Baseline Hb <LLN (yes vs. no)		0.940 (0.484–1.823) *p*=0.8543	3.595 (1.315–9.824) *p*=0.0126	0.940	3.378

Abbreviations: *CI* confidence interval, *Hb* haemoglobin, *HR* hazard ratio, *LLN* lower limit of normal

[a] *p*-values from 2-sided Wald test.

[b] Hazard ratio for each covariate in the axitinib titration arm is derived from the multiplicity of the covariate hazard ratio and the interaction hazard ratio.
